# Association between *TERT* promoter mutations and clinical behaviors in differentiated thyroid carcinoma: a systematic review and meta-analysis

**DOI:** 10.1007/s12020-019-02117-2

**Published:** 2019-10-26

**Authors:** Jing Yang, Yanping Gong, Shuping Yan, Hui Chen, Siqin Qin, Rixiang Gong

**Affiliations:** 1 Thyroid and Parathyroid Surgery Center, West China Hospital, Sichuan University, No. 37 Guo Xue Xiang, Chengdu, 610041 Sichuan China; 2 West China School of Medicine, Sichuan University, No. 37 Guo Xue Xiang, Chengdu, 610041 Sichuan China; 3 Department of General Surgery, the Second People’s Hospital of Deyang City, No. 340 West Minjiang Road, Deyang, 618000 Sichuan China

**Keywords:** *TERT*, DTC, PTC, FTC, Clinical behaviors

## Abstract

**Background:**

The association between telomerase reverse transcriptase (*TERT*) promoter mutations and some clinical behaviors in thyroid cancer remains controversial and requires additional investigation. This study aimed to evaluate the association between *TERT* promoter mutations and clinical behaviors (including clinicopathological features and prognosis) in differentiated thyroid carcinomas (DTC).

**Methods:**

We performed an up-to-date systematic review and current comprehensive meta-analysis. We searched three electronic databases for relevant studies. We used fixed- or random-effect models to calculate pooled estimated odds ratios (ORs) or standardized mean differences (SMDs) and corresponding 95% confidence intervals (CIs).

**Results:**

We included 51 eligible studies incorporating 11,382 cases. Average frequencies of *TERT* promoter mutations in DTC, papillary (PTC), and follicular (FTC) thyroid carcinomas were 10.9%, 10.6%, and 15.1%, respectively. In DTC and PTC, *TERT* promoter mutations were significantly associated with sex, age, tumor size, vascular invasion, extrathyroidal extension, lymph node and distant metastases, advanced tumor, nodes, and metastasis (TNM) stage, persistence/recurrence, and disease-specific mortality. In FTC, *TERT* promoter mutations were significantly associated with age, distant metastases, advanced TNM stage, persistence/recurrence, and disease-specific mortality.

**Conclusions:**

*TERT* promoter mutations could be considered as biomarkers assisting in risk stratification, prognostic prediction, and individualizing therapeutic options for DTC (PTC and FTC).

## Introduction

Thyroid cancer is the most common endocrine tumor, and its incidence is increasing worldwide [1]. Among the follicular cell-derived thyroid cancers, papillary (PTC) and follicular (FTC) types are well-differentiated and classified as differentiated thyroid carcinomas (DTC), the most common thyroid malignancy [2]. Although most DTCs have a favorable clinicopathological behavior and improved prognosis, a small proportion of cases show aggressive behavior with adverse outcome [3]. Some clinicopathological factors, such as old age, large tumor size, or distant metastasis, have been associated with poor outcomes of DTC [3, 4]. However, these factors are not entirely reliable in predicting tumor recurrence or cancer-related mortality in DTC. For precise risk stratification, several studies have been conducted to identify some molecular markers in PTC and FTC, such as genetic alterations.

The telomerase reverse transcriptase (*TERT*) gene is a 35 kb gene located on chromosome 5, which contains 16 exons and a promoter region of 330 base pairs (bp) [5]. Mutations in the coding regions of the gene are rare [6]. Interestingly, mutations in the promoter region have been described recently in melanomas through whole-genome sequencing [7, 8], and also quickly found in other human cancers, such as glioma, myxoid liposarcoma, and hepatocellular, urothelial (bladder) [9–11], as well as thyroid [11, 12] carcinomas. Two common recurrent *TERT* promoter mutations in human cancer are located at two hotspots: chr5, 1,295,228 COT (C228T) and 1,295,250 COT (C250T), corresponding to the positions −124 and −146 bp, respectively, upstream of the ATG start site [7, 8]. Both mutations generate a consensus binding site (GGAA) in the *TERT* promoter region for E-26 (ETS) transcription factors, which has been shown to confer *TERT* promoter-enhanced transcriptional activities [7, 8, 13].

Since the *TERT* promoter mutations were reported first by Liu et al. in thyroid cancer [12], subsequently, a large number of studies have reported the association between *TERT* promoter mutations and clinical behaviors (including pathological features and prognosis) in thyroid cancer, especially in DTC [14–23]. However, some results remain controversial and require additional investigation. Therefore, we performed an up-to-date systematic review and current comprehensive meta-analysis to evaluate the association of *TERT* promoter mutation and clinical parameters in DTC. These clinical parameters included: mean age, gender, mean tumor size, multifocality, vascular invasion, extrathyroidal extension, lymph node metastasis (LNM), distant metastases, advanced tumor, nodes, and metastasis (TNM) stage, persistence/recurrence, and disease-specific mortality. The meta-analysis results could provide new insight into the biology of *TERT* promoter mutations and understanding of the clinical significance of these mutation carriers, and offer implications for the design of clinical trials, particularly those of anticancer targeted agents for the *TERT* promoter in aggressive thyroid cancers.

## Materials and methods

### Selection criteria

We extensively searched for studies that examined the associations of *TERT* promoter mutations and clinical parameters in DTC (PTC and/or FTC). In some articles, PTC and FTC were independently analyzed, whereas in other articles, PTC and FTC were synthesized as DTC for analysis. Therefore, DTC (PTC and FTC together) was selected as a separate group for meta-analysis, and PTC and FTC were selected as separate subgroups for meta-analysis. The inclusion criteria for selecting studies were articles published in English from inception to December 31, 2018, clinical parameters with detailed data on DTC, PTC, and FTC included from articles on different types of thyroid carcinoma (PTC, FTC, medullary, differentiated, poorly-differentiated, and anaplastic), only studies analyzing at least one category of clinical data, and, when multiple articles were published by the same authors, the newest/most informative single article was selected. We excluded articles on thyroid cancer subtypes other than DTC, PTC, or FTC; review articles or meta-analyses without original data; single or pure case reports; posters, conference papers, theses, or books; absent or inappropriately reported clinical data; animal or cell lines studies; and duplicated articles. Any disagreements between two reviewers were solved by discussion and consensus.

### Search strategy

We searched PubMed, Embase, and Web of Science databases to identify all potential clinical studies from inception to December 31, 2018. We selected English language articles with a combination of the following search terms: TS = ([TERT OR “telomerase reverse transcriptase”] AND promoter AND thyroid). In addition, we searched for potential studies by reviewing the citations within the included studies and reviews. All procedures strictly followed the recommendation of Preferred Reporting Items for Systematic Review and Meta-analysis statement [24].

### Articles screening and data extraction

Two investigators (Yanping Gong and Jing Yang) used the EndNote (Thompson Reuters, PA, US) reference tool to screen and select articles independently. The full-text of all relevant studies was downloaded consecutively and screened independently by two reviewers. The variables extracted by two investigators independently based on the same rules were first author, publication year, country, number of patients by *TERT* promoter, number of males or females, mean age at diagnosis, mean tumor size, TNM stages, LNM, extrathyroidal extension, distant metastasis, persistence/recurrence, and disease-specific mortality. We carefully avoided any duplication of data by examining the names of all the authors and the medical centers involved in each publication. Overlapping articles or data and articles unrelated to our questions were excluded. In cases of insufficient or unpublished data, we tried to obtain potential further data by contacting the authors via email. Studies in which clinical parameter data were not provided in the original study or via email were further excluded from the final analyses.

### Quality assessment and risk of bias analysis

The quality of the included studies was evaluated according to the Newcastle–Ottawa scale (NOS) comprising four stars for selection, two stars for comparability, and three stars for outcome. Two reviewers independently awarded the stars for cohort or case-control studies (maximum nine stars) based on a developed checklist [25]. Studies awarded at least six stars were considered moderate to high-quality and those with a NOS value of less than six were regarded as low-quality.

### Data analyses and statistical methods

STATA 14 software (Stata Corporation, College Station, TX, USA) was used for all statistical analyses, including the calculation of the summary odds ratio (OR) or standardized mean difference (SMD) with a 95% confidence interval (95% CI), using a random- or fixed-effect model for all the analyses. The choice of each individual statistical method depended on whether the measured event was dichotomous or continuous, whereas the choice of a random- or fixed-effect model depended on the tests for heterogeneity. We assessed heterogeneity using the *χ*^2^ test of heterogeneity and the *I*^2^ measure of inconsistency. If heterogeneity in the *χ*^2^ test or *I*^2^ measure showed a *P* value of <0.10 or >50%, respectively, the random-effect model was chosen, otherwise the fixed-effect model was used. The 95% CI was constructed around the effect size to establish its significance. We conducted a sensitivity analysis to estimate the effects of the remaining studies without the larger one’s effect to examine the strength of the outcome. Funnel plot analysis and Egger’s test was used to assess the potential for publication bias.

For the OR of dichotomous events, if the 95% CI of an OR included 1, the two groups were not considered statistically different, otherwise they were considered significant. For continuous event SMD, if the 95% CI crossed the null point (zero), then the possibility that the difference should be attributed to chance could not be ruled out. When the null point fell outside the 95% CI of an SMD, the observed difference was considered statistically significant. Funnel plot and/or Egger’s regression test was done to assess further the presence of publication bias and calculated by Meta-Essentials: Workbook for meta-analysis [25]. *P* < 0.05 was considered statistically significant.

## Result

### Search results and quality assessment

**Fig. 1:**
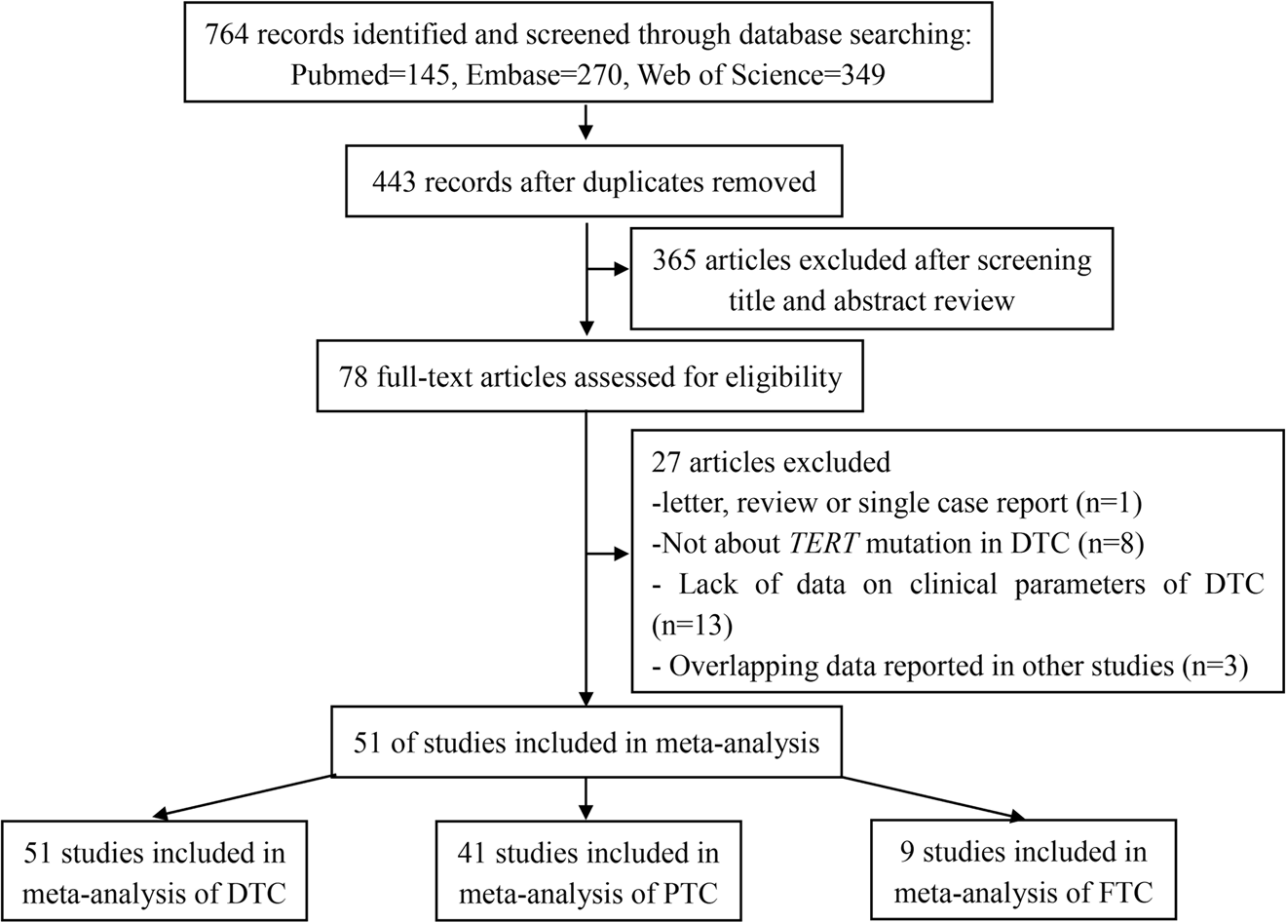
Flowchart of section process. *TERT*, telomerase reverse transcriptase; DTC, differentiated thyroid carcinoma; PTC, papillary thyroid carcinoma; FTC: follicular thyroid carcinoma

Figure 1 shows the flowchart of the literature research. Initially, 764 studies were included. After removal of duplicates, 443 studies remained. Then, 365 studies were excluded after reviewing the titles and abstracts; 78 full-text studies were evaluated further in detail, and ultimately, 51 studies contributed 11,382 cases with DTC to the meta-analysis for analyzing the correlation between *TERT* promoter mutation and clinical behaviors in DTC. Of the 51 studies included, 41 and 9 investigated the association between *TERT* promoter mutation and clinical behaviors in PTC and FTC, respectively. In DTC, the frequencies of *TERT* promoter mutation ranged from 2.1 to 75%, and overall average frequency was 10.9% (1239/11,382). When calculated in PTC and FTC separately, the average frequencies of *TERT* promoter mutation were 10.6% (1027/9653) and 15.1% (79/522), respectively. The NOS tool was used to assess the quality of the included studies, with five to nine stars awarded to each study. Table 1 describes the characteristics of the included studies and the details of NOS stars given in the meta-analysis.

**Table 1:** A summary of 51 studies included in the meta-analysis

Study	Country	No. of cases	Histotype	No. of *TERT* promoter mutation				Clinical parameters	Quality (NOS)
				Both (%)	C228T	C250T	Other		
Vinagre et al. [11]	Portugal	233	PTC + FTC + HCC	23 (9.9)	19	3	1	Gender, age, tumor size, LNM	6
Liu et al. [14]	Sweden	87	PTC + FTC	21 (24.1)	19	2	NA	Gender, age, tumor size, EE, LNM, DSM	9
Liu et al. [15]	USA	408	PTC	46 (11.3)	39	7	NA	Gender, age, tumor size, EE, LNM, TNM stage	5
Melo et al. [16]	Portugal	402	PTC + FTC	37 (9.2)	NA	NA	NA	Gender, age, tumor size, EE, VI, LNM, DM, TNM stage, persistence, DSM	8
Wang et al. [26]	Sweden	52	FTC	9 (17.3)	8	1	NA	Gender, age, DSM	8
Xing et al. [17]	USA	507	PTC	61 (12.0)	61	0	NA	Gender, age, multifocality, EE, VI, LNM, DM, TNM stage, recurrence	6
de Biase et al. [27]	Italy	404	mPTC	19 (4.7)	11	8	NA	Gender, age, tumor size, multifocality, EE, LNM, TNM stage, persistence/recurrence	8
Dettmwe et al. [64]	Switzerland	110	TCPTC	8 (7.3)	5	3	NA	Recurrence/DSM	5
Gandolfi et al. [28]	Italy	121	PTC	21 (17.4)	12	5	4	Gender, age, VI, LNM, DM, TNM stage, persistence/recurrence, DSM	8
George et al. [29]	USA	242	p/rPTC	77 (31.8)	77	0	NA	Gender, LNM, DM, TNM stage, DSM	8
Lee et al. [61]	Korea	137	cPTC	9 (6.6)	9	0	NA	EE, LNM	5
Muzza et al. [30]	Italy	240	PTC + FTC	30 (12.5)	24	6	NA	Gender, multifocality, EE, LNM, TNM stage, persistence/recurrence	9
Qasem et al. [31]	Saudi Arabia	243	PTC	26 (10.7)	20	6	NA	Gender, multifocality, EE, VI, LNM, DM, TNM stage, persistence/recurrence	9
Bae et al. [32]	Korea	222	PTC + FTC	12 (5.4)	10	2	NA	Gender, age, tumor size, EE, LNM, DM, TNM stage	5
Bullock et al. [18]	Australia	80	PTC	11 (13.8)^a^	8	4	NA	Gender, LNM, TNM stage, Persistence/recurrence, DSM	8
Crescenzi et al. [59]	Italy	31	PTC + FTC	3 (9.7)	3	0	NA	Multifocality, LNM	5
Jeon et al. [33]	Korea	35	cPTC	1 (2.9)	1	0	NA	Gender, LNM, recurrence	8
Jin et al. [19]	China	653	PTC	27 (4.1)	23	4	NA	Gender, age, tumor size, multifocality, EE, LNM, TNM stage	5
Kim et al. [34]	Korea	393	PTC + FTC	43 (10.9)	39	4	NA	Gender, multifocality, EE, LNM, DM, TNM stage	8
Lee et al. [35]	Korea	207	PTC	30 (14.5)	30	0	NA	Gender, age, tumor size, multifocality, EE, LNM, TNM stage, recurrence	8
Liu et al. [68]	USA	1051	PTC	130 (12.4)	NA	NA	NA	DSM	8
Myung et al. [36]	Korea	74	PTC	13 (17.6)	11	2	NA	Gender, age, tumor size, multifocality, EI, LNM, TNM stage, persistence/recurrence	8
Nasirden et al. [37]	Japan	137	PTC	8 (5.8)	8	0	NA	Gender, multifocality, EE, VI, LNM, TNM stage, persistence/recurrence	8
Sohn et al. [38]	Korea	17	PTC + FTC	12 (70.6)	11	1	NA	Gender, age, LNM, DM, TNM stage	5
Song et al. [39]	Korea	551	PTC + FTC	25 (4.5)	21	4	NA	Gender, MA, EE, LNM, DM, TNM stage, persistence/recurrence, DSM	7
Sun et al. [40]	China	434	PTC	19 (4.4)	18	1	NA	Gender, age, multifocality, LNM, TNM stage	5
Boaventura et al. [41]	Portugal	27	WDTC	4 (14.8)	4	0	NA	Gender, MA	6
Hahn et al. [42]	Korea	150	PTC	11 (7.3)	11	0	NA	Gender, multifocality, EE, TNM stage, recurrence, DSM	9
Kim et al. [65]	Korea	327	PTC + FTC	30 (9.2)	27	3	NA	recurrence	9
Marques et al. [60]	Portugal	54	PTC + FTC	5 (9.3)	4	1	NA	Multifocality, EE, VI, LNM, DM, recurrence, DSM	8
Matsuse et al. [43]	Japan	357	PTC	36 (10.1)	33	3	NA	Gender, EE, LNM, DM, TNM stage, recurrence	8
Melo et al. [44]	Portugal	195	PTC + FTC	27 (13.8)	NA	NA	NA	Gender, age, tumor size, EE, VI, LNM, DM, TNM stage	5
Morandi et al. [66]	Italy	18	HVPTC	8 (44.4)	4	3	1	Persistence/recurrence, DSM	8
Oishi et al. [45]	Japan	85	aPTC	11 (12.9)	8	3	NA	Gender, age, tumor size, EE, LNM, DM, TNM stage	5
Shen et al. [46]	USA	388	PTC	39 (10.1)	30	8	1	Gender, multifocality, EE, LNM, DM, TNM stage, recurrence, DSM	8
Song et al. [20]	Korea	120	FTC	7 (5.8)	6	1	NA	Gender, age, tumor size, multifocality, EE, VI, LNM, DM, TNM stage, persistence/recurrence, DSM	9
Xu et al. [47]	USA	8	PTC + HCC	6 (75.0)	NA	NA	NA	Gender, age, tumor size, multifocality, DSM	8
Yang et al. [48]	China	66	PTC + FTC	15 (22.7)	13	2	NA	Gender, age, tumor size, multifocality, EE, LNM	6
Argyropoulou et al. [49]	Greek	59	PTC	2 (3.4)	NA	NA	NA	Gender, EE, LNM	5
Bu [50]	Saudi Arabia	927	PTC	167 (18.0)^a^	144	24	NA	Gender, EE, VI, LNM, DM, TNM stage	8
Colombo et al. [51]	Italy	208	PTC	49 (23.6)	46	3	NA	Gender, age, multifocality, EE, LNM	9
Gandolfi et al. [63]	Italy	126	PTC	21 (16.7)	13	8	NA	DM	5
Insilla et al. [52]	Italy	145	PTC	9 (6.2)	8	1	NA	Gender, EE, LNM,	6
Liang et al. [53]	China	355	PTC	7 (2.1)	5	2	NA	Gender, age, tumor size, LNM	7
Paulsson et al. [67]	Sweden	94	FTC	19 (20.2)	NA	NA	NA	Recurrence	7
Poma et al. [57]	Italy	20	FTC	2 [10]	NA	NA	NA	Age, tumor size, VI	8
Ren et al. [54]	China	342	PTC	12 (3.5)	10	2	NA	Gender, age, tumor size, multifocality, EE, LNM, TNM stage	6
Rusinek et al. [55]	Poland	189	PTC	22 (11.6)	13	3	6	Gender, multifocality, VI, LNM	7
Tavares et al. [62]	Portugal	11	PTC + FTC	1 (9.1)	NA	NA	1	LNM, DM, persistence/recurrence	5
Watutantrige-Fernando et al. [56]	Italy	24	HVPTC	3 (12.5)	2	0	1	Gender, age, tumor size, LNM, DM, TNM stage, persistence/recurrence	8
Wong et al. [58]	USA	16	TCVPTC	5 (31.−3)	NA	NA	NA	Tumor size, EE, LNM, recurrence	5

*No*. number, NA not applicable/not available, *TERT* telomerase reverse transcriptase, *VI* vascular invasion, *EE* extrathyroidal extension, *LNM* lymph node metastasis, *DM* distant metastasis, *DSM* disease-specific mortality, *PTC* papillary thyroid carcinoma, *FTC* follicular thyroid carcinoma, *HCC* Hürthle cell carcinoma, *WDTC* well-differentiated thyroid carcinoma, *mPTC* papillary thyroid microcarcinomas, *p/rPTC* persistent/recurrent PTC, *cPTC* conventional/classic PTC, *aPTC* adult PTC, *HVPTC* Hobnail variant of PTC, *TCVPTC* tall cell variant of PTC, *NOS* Newcastle–Ottawa scale

^a^one case harbored double mutations

Among 51 studies of overall DTC, 39 [11, 14–20, 26–56], 26 [11, 14–17, 19, 20, 26–28, 32, 35, 36, 38–41, 44, 45, 47, 48, 51, 53, 54, 56, 57], 19 [11, 14–16, 19, 20, 27, 32, 35, 36, 44, 45, 47, 48, 53, 54, 56–58], 20 [17, 19, 20, 27, 30, 31, 34–37, 40, 42, 46–48, 51, 54, 55, 59, 60], 11 [16, 17, 20, 28, 31, 37, 44, 50, 55, 57, 60], 29 [14–17, 19, 20, 27, 30–32, 34–37, 39, 42–46, 48–52, 54, 58, 60, 61], 39 [11, 14–20, 27–35, 37–40, 43–46, 48–56, 58–62], 19 studies [16, 17, 20, 28, 29, 31, 32, 34, 38, 39, 43–47, 56, 60, 62, 63], 27 [15–20, 27–32, 34–40, 42–46, 50, 54, 56], 24 [16–18, 20, 27, 28, 30, 31, 33, 35–38, 42, 43, 46, 56, 58, 60, 62, 64–67], and 14 [14, 16, 18, 20, 26, 28, 29, 39, 42, 46, 47, 60, 66, 68] studies were analyzed for the associations between *TERT* promoter mutation and gender, mean age, mean tumor size, multifocality, vascular invasion, extrathyroidal extension, LNM, distant metastasis, advanced TNM stage, persistence/recurrence, and disease-specific mortality, respectively. Among 41 studies of PTC, 32 [11, 14–19, 27–31, 33, 35–40, 42, 43, 45–47, 49–56], 19 [11, 14–17, 19, 27, 28, 35, 36, 38–40, 45, 47, 51, 53, 54, 56], 14 [11, 14–16, 19, 27, 35, 36, 45, 47, 53, 54, 56, 58], 16 [17, 19, 27, 30, 31, 35–37, 40, 42, 46, 47, 51, 54, 55, 59], 7 [16, 17, 28, 31, 37, 50, 55], 23 [14–17, 19, 27, 30, 31, 35–38, 42, 43, 45, 46, 49–52, 54, 58, 61], 32 [11, 14–19, 27–31, 33, 35, 37, 38, 40, 43, 45, 46, 49–56, 58, 59, 61, 62], 15 [16, 17, 28, 29, 31, 32, 38, 39, 43, 45, 46, 50, 56, 62, 63], 24 [15–19, 27–31, 35–40, 42, 43, 45, 46, 50, 51, 54, 56], 19 [17, 18, 27, 28, 30, 31, 33, 35–37, 39, 42, 43, 46, 56, 58, 62, 64, 66], and 10 [14, 16, 18, 28, 29, 39, 42, 46, 66, 68] studies were analyzed for the abovementioned associations, respectively. Among 9 studies of FTC, 7 [11, 14, 16, 20, 26, 30, 38], 7 [11, 14, 16, 20, 26, 38, 57], 4 [14, 16, 20, 57], 2 [20, 30], 3 [16, 20, 57], 3 [16, 20, 30], 4 [14, 16, 20, 30], 3 [16, 20, 38], 3 [16, 20, 30], 3 [20, 30, 67], and 4 [14, 16, 20, 26] studies were analyzed for the abovementioned associations, respectively.

Fixed-effects models were used for analysis of gender, multifocality, vascular invasion, LNM, persistence/recurrence, and disease-specific mortality in the DTC studies, and in the analysis of gender, multifocality, vascular invasion, LNM, persistence/recurrence, and disease-specific mortality in the PTC studies, whereas random-effects models were chosen for the other analyses. Fixed-effects model was used in the analysis of all the clinical parameters in the FTC studies.

### Association between *TERT* promoter mutations and clinical parameters in DTC

**Fig. 2:**
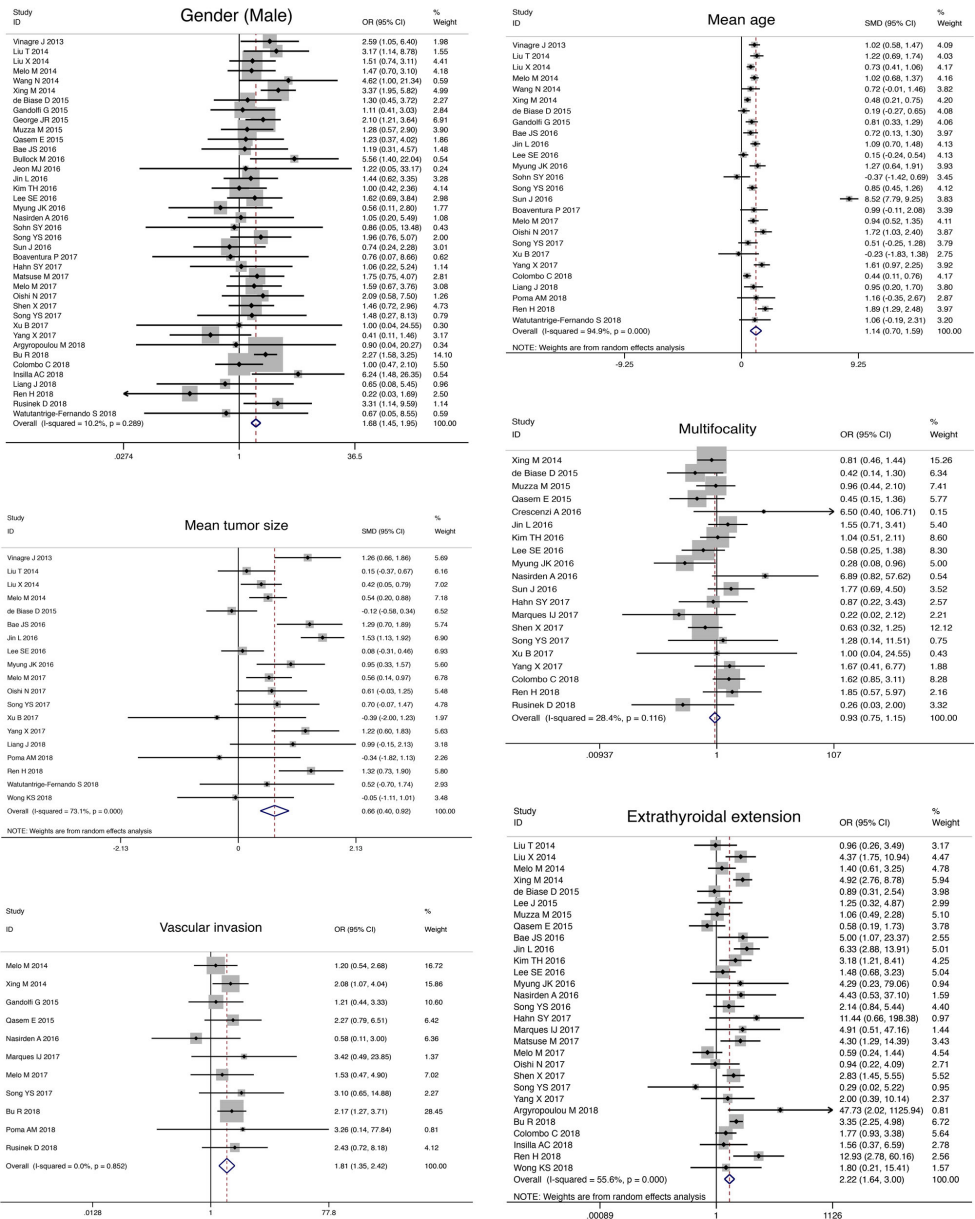

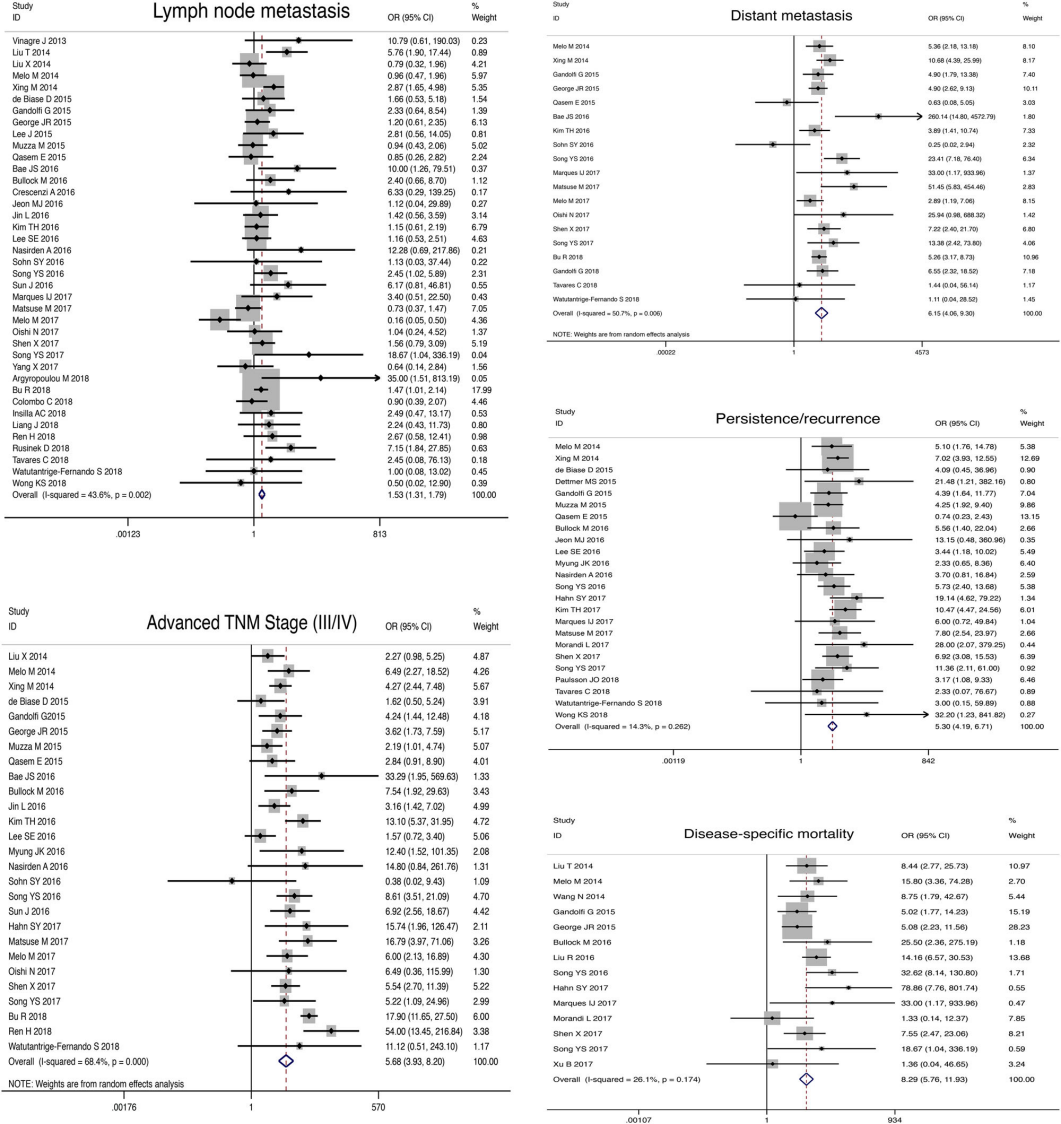
Forest plot showing the association of *TERT* promoter mutations with clinical parameters in DTC

*TERT* promoter mutations tended to present in older patients (SMD, 1.14; 95% CI, 0.70–1.59; *P* < 0.05) and with larger tumor size (SMD, 0.66; 95% CI, 0.40–0.92; *P<* 0.05; Table 2). Besides, *TERT* promoter mutations were associated with male gender (OR, 1.68; 95% CI, 1.45–1.95; *P* < 0.05), vascular invasion (OR, 1.81; 95% CI, 1.35–2.42; *P* < 0.05), extrathyroidal extension (OR, 2.22; 95% CI, 1.64–3.00; *P* < 0.05), LNM (OR, 1.53; 95% CI, 1.31–1.79; *P* < 0.05), distant metastasis (OR, 6.15; 95% CI, 4.06–9.30; *P* < 0.05), and advanced TNM stage (OR, 5.68; 95% CI, 3.93–8.20; *P* < 0.05). *TERT* promoter mutations were also associated with adverse outcomes, including tumor persistence/recurrence (OR, 5.30; 95% CI, 4.19–6.71; *P* < 0.05) and disease-specific mortality (OR, 8.29; 95% CI, 5.76–11.93; *P* < 0.05). However, *TERT* promoter mutations were not associated with multifocality (OR, 0.93; 95% CI, 0.75–1.15; *P=* 0.478). Forest plots concerning the association of *TERT* promoter mutation and these clinical parameters are shown in Fig. 2.

**Table 2:** Meta-analyses of association between clinical behaviors and *TERT* promoter mutation in DTC, PTC and FTC

Clinical parameters	No. of studies	No. of cases	Heterogeneity test			Effects model selection	OR /SMD (95 % CI)	Combined effect test		Statistical significance	Egger’s test
			*χ*^2^	*P*	*I*^2^			*Z*	*P*		*P*
**DTC**											
Gender (Male)	39	9226	42.33	0.289	10.2%	Fixed	1.68 (1.45, 1.95)	6.88	0.000	Yes	0.261
Mean age	26	5732	493.94	0.000	94.9%	Random	1.14 (0.70, 1.59)	5.02	0.000	Yes	0.195
Mean tumor size	19	3541	66.82	0.000	73.1%	Random	0.66 (0.40, 0.92)	4.96	0.000	Yes	0.999
Multifocality	20	4745	26.53	0.116	28.4%	Fixed	0.93 (0.75, 1.15)	0.71	0.478	No	0.151
Vascular invasion	11	2092	5.54	0.852	0.0%	Fixed	1.81 (1.35, 2.42)	3.99	0.000	Yes	0.688
Extrathyroidal extension	29	7224	63.04	0.000	55.6%	Random	2.22 (1.64, 3.00)	5.19	0.000	Yes	0.190
Lymph node metastasis	39	8374	67.42	0.002	43.6%	Fixed	1.53 (1.31,1.79)	5.32	0.000	Yes	0.001
Distant metastasis	19	4608	36.51	0.006	50.7%	Random	6.15 (4.06, 9.30)	8.60	0.000	Yes	0.079
Advanced TNM Stage (III/IV)	27	7334	82.16	0.000	68.4%	Random	5.68 (3.93, 8.20)	9.26	0.000	Yes	0.827
Persistence/recurrence	24	4245	26.85	0.262	14.3%	Fixed	5.30 (4.19, 6.71)	13.83	0.000	Yes	0.105
Disease-specific mortality	14	3267	17.59	0.174	26.1%	Fixed	8.29 (5.76, 11.93)	11.37	0.000	Yes	0.146
**PTC**											
Gender (Male)	32	7824	35.36	0.270	12.3%	Fixed	1.80 (1.53, 2.11)	7.14	0.000	Yes	0.471
Mean age	19	4742	485.60	0.000	96.3%	Random	1.25 (0.66, 1.85)	4.14	0.000	Yes	0.162
Mean tumor size	14	2842	57.77	0.000	77.5%	Random	0.60 (0.27, 0.94)	3.51	0.000	Yes	0.705
Multifocality	16	4052	24.20	0.062	38.0%	Fixed	0.92 (0.73, 1.16)	0.73	0.467	No	0.151
Vascular invasion	7	1742	6.73	0.347	10.8%	Fixed	1.71 (1.24, 2.35)	3.26	0.001	Yes	0.171
Extrathyroidal extension	23	6019	48.34	0.001	54.5%	Random	2.37 (1.71, 3.27)	5.19	0.000	Yes	0.204
Lymph node metastasis	32	7105	42.25	0.086	26.6%	Fixed	1.64 (1.38, 1.95)	5.62	0.000	Yes	0.007
Distant metastasis	15	3684	33.59	0.002	58.3%	Random	6.49 (3.82, 11.01)	6.93	0.000	Yes	0.103
Advanced TNM Stage (III/IV)	24	6355	93.91	0.000	75.5%	Random	4.82 (3.12, 7.43)	7.11	0.000	Yes	0.941
Persistence/recurrence	19	3232	23.18	0.184	22.3%	Fixed	4.97 (3.78, 6.53)	11.49	0.000	Yes	0.118
Disease-specific mortality	10	2808	15.30	0.083	41.2%	Fixed	8.29 (5.57, 12.34)	10.42	0.000	Yes	0.128
**FTC**											
Gender (male)	7	403	10.01	0.124	40.0%	Fixed	1.17 (0.64, 2.15)	0.51	0.607	No	NA
Mean age	7	367	3.66	0.722	0.0%	Fixed	0.72 (0.41, 1.03)	4.54	0.000	Yes	NA
Mean tumor size	4	235	3.05	0.384	1.6%	Fixed	0.14 (-0.26, 0.54)	0.67	0.503	No	NA
Multifocality	2	178	0.18	0.669	0.0%	Fixed	0.89 (0.19, 4.22)	0.15	0.880	No	NA
Vascular invasion	3	193	0.40	0.819	0.0%	Fixed	2.28 (0.75, 6.90)	1.46	0.144	No	NA
Extrathyroidal extension	3	227	2.70	0.260	25.9%	Fixed	1.57 (0.59, 4.15)	0.91	0.363	No	NA
Lymph node metastasis	4	261	3.32	0.345	9.6%	Fixed	1.96 (0.72, 5.37)	1.31	0.190	No	NA
Distant metastasis	3	159	0.99	0.611	0.0%	Fixed	24.29 (6.30, 93.58)	4.63	0.000	Yes	NA
Advanced TNM Stage (III/IV)	3	207	0.45	0.798	0.0%	Fixed	5.10 (1.81, 14.35)	3.08	0.002	Yes	NA
Persistence/recurrence	3	272	1.67	0.425	0.0%	Fixed	4.59 (2.08, 10.13)	3.77	0.000	Yes	NA
Disease-specific mortality	4	278	0.94	0.815	0.0%	Fixed	9.28 (3.35, 25.70)	4.28	0.000	Yes	NA

*No*. number, *OR* odds ratio, *SMD* standardized mean difference, *CI* confidence interval, *NA* not applicable/not available

### Association between *TERT* promoter mutations and clinical parameters in PTC

*TERT* promoter mutation tended to present in older patients (SMD 1.25; 95% CI, 0.66–1.85; *P* < 0.05) and with larger tumor size (SMD, 0.60; 95% CI, 0.27–0.94; *P* < 0.05; Table 2). Besides, *TERT* promoter mutations were associated with male gender (OR, 1.80; 95% CI, 1.53–2.11; *P* < 0.05), vascular invasion (OR, 1.71; 95% CI, 1.24–2.35; *P* < 0.05), extrathyroidal extension (OR, 2.37 95% CI, 1.71–3.27, *P* < 0.05), LNM (OR, 1.64; 95% CI, 1.38–1.95; *P* < 0.05), distant metastasis (OR, 6.49; 95% CI, 3.82–11.01; *P* < 0.05), and advanced TNM stage (OR, 4.82; 95% CI, 3.12–7.43; *P* < 0.05). *TERT* promoter mutations were also associated with adverse outcomes including tumor persistence/recurrence (OR, 4.97; 95% CI, 3.78–6.53; *P* < 0.05) and disease-specific mortality (OR, 8.29; 95% CI, 5.57–12.34; *P* < 0.05). However, *TERT* promoter mutations were not associated with multifocality (OR, 0.92; 95% CI, 0.73–1.16; *P=* 0.890). Supplementary Fig. 1 shows forest plots concerning the association of *TERT* promoter mutation and these clinical parameters.

### Association between *TERT* promoter mutation and clinical behaviors in FTC

*TERT* promoter mutation tended to present in older patients (SMD 0.72; 95% CI, 0.41–1.03; *P* < 0.05; Table 2). Besides, *TERT* promoter mutations were associated with distant metastasis (OR, 24.29; 95% CI, 6.30–93.58; *P* < 0.05) and advanced TNM stage (OR, 5.10; 95% CI, 1.81–14.35; *P* < 0.05). *TERT* promoter mutations were also associated with adverse outcomes including tumor persistence/recurrence (OR, 4.59; 95% CI, 2.08–10.13 *P* < 0.05) and disease-specific mortality (OR, 9.28; 95% CI, 3.35–25.70; *P* < 0.05). However, *TERT* promoter mutations were not associated with gender (OR, 1.17; 95% CI, 0.64–2.15; *P=* 0.607), tumor size (SMD, 0.14; 95% CI, −0.26 to 0.54; *P=* 0.503), multifocality (OR, 0.89; 95% CI, 0.19–4.22; *P=* 0.880), vascular invasion (OR, 2.28; 95% CI, 0.75–6.90; *P=* 0.144), extrathyroidal extension (OR, 1.57; 95% CI, 0.59–4.15, *P=* 0.363), and LNM (OR, 1.96; 95% CI, 0.72–5.37; *P=* 0.190). Supplementary Fig. 2 shows forest plots concerning the association of *TERT* promoter mutation and these clinical parameters.

### Heterogeneity assessment

We used sensitivity analysis by removing each of the included studies to find which studies influenced the degree of heterogeneity. All the significant pooled results following the leave-one-out method remained unaffected.

### Publication bias

Funnel plot observation did not show strong evidence of publication bias among the set of studies. Except for the analysis of LNM in DTC and PTC, most of Egger’s regression test of all the effects did not suggest any evidence of publication bias (data shown in Table 2). When we simultaneously eliminated four studies by Vinagre et al. [11], Nasirden et al. [37], Song et al. [48], and Argyropoulu et al. [49] on DTC and eliminated four studies by Vinagre et al. [11], Liu et al. [14], Nasirden et al. [37], and Argyropoulu et al. [49] on PTC for analysis of LNM, these publication biases disappeared and the significant pooled results remained unaffected. In FTC, Egger’s regression test was not performed because of the small numbers of included studies.

## Discussion

Many somatic genetic alterations, including those in *BRAF, HRAS, KRAS, NRAS, PTEN*, and *HER1*, have had fundamental roles in the tumorigenesis of thyroid carcinoma. Recently, the close association of *TERT* promoter somatic mutations with tumorigenesis is widely recognized also. Since Liu et al. first directly investigated the diagnostic and prognostic potentials of preoperative testing of thyroid fine-needle aspiration biopsy (FNAB) specimens for the *TERT* promoter mutations in thyroid cancer [12], a significant interest in *TERT* promoter mutations (mainly C228T and C250T) focused on the frequency of these mutations in different subtypes of thyroid cancer and their association with clinicopathological features and outcomes of thyroid cancer has accumulated. A large number of publications have been generated over the last ~5 years. It is not controversial that the *TERT* promoter mutations are associated with poor outcome. However, the associations between *TERT* promoter mutations and some clinicopathological features remain discrepant. Our study aimed to explore the influence of *TERT* promoter somatic mutations on the clinicopathological features and prognosis of DTC via an updated meta-analysis. In our study, the results of meta-analyses of PTC were in line with those of DTC, but some results of FTC not with those of DTC. This may be attributed to the fact that the sample capacity of DTC has derived mainly from PTC cases, but lesser from FTC cases. To the best of our knowledge, our study is the up-to-date meta-analysis evaluating the association between *TERT* promoter mutation and clinical behaviors in PTC, and it is the first meta-analysis independently investigating the association between *TERT* promoter mutations and clinical behaviors in FTC.

Although two similar meta-analyses had been performed to investigate the association of *TERT* promoter mutations with clinicopathological features and prognosis of PTC, their literature searches were performed in November 2015 and the numbers of included studies were small (eight and ten studies respectively) [21, 22]. However, our literatures searches were performed from the inception to December 31, 2018, and 51 studies were included in our study, 41 of which investigated the association between *TERT* promoter mutation and clinical behaviors in PTC. Besides, our study included the overall clinicopathological and prognostic parameters. In the present study, the overall average frequency of *TERT* promoter mutations was 10.9% in DTC, which was close to that in PTC (10.6%). This is because the number of PTC cases was greater than that of FTC (9653:522). In some publications, the frequency of *TERT* promoter mutations was reported to be as high as 75% and as low as 2.1%. This can possibly be attributed to small sample size. In the previous two studies, the average frequencies of *TERT* promoter mutation in PTC were 10.3% and 10.1%, respectively [21, 22]. In our study, the average frequency in PTC was 10.6%, which was similar to the previously reported results [21, 22]. Almost all previous studies reported no collective prevalence of *TERT* promoter mutations in normal thyroid parenchyma or benign thyroid lesions [11, 12, 15, 16, 30, 69], such as nodular goiter (hyperplasia lesions), diffuse toxic goiters, lymphocytic (Hashimoto’s) thyroiditis, and follicular thyroid adenomas (FTA). The *TERT* promoter mutations were sporadically reported only in two FTAs [26, 70]. Therefore, we postulated that *TERT* promoter mutation may have an important role in preoperative diagnosis of thyroid carcinoma, especially for patients with indeterminate cytology on FNAB.

Some aggressive clinicopathological characters, for example, male gender, larger tumor size, extrathyroidal extension, LNM, distant metastasis, and advanced TNM stage, were correlated with poor prognostic features, such as persistence/recurrence and disease-specific mortality in previous studies [18, 23]. Previous meta-analyses suggested that *TERT* promoter mutations were associated with these aggressive clinicopathological characteristics [21, 22], which were mainly in accordance with the results of our meta-analysis. Our findings indicated that *TERT* promoter mutations were more likely to be present in male patients, and those of older age, with larger tumor size, and strongly-associated vascular invasion, extrathyroidal extension, LNM, distant metastasis, and advanced TNM stage in PTC. However, the previous two meta-analyses found that the association of *TERT* promoter mutations with vascular invasion was not significant (*P* = 0.20 and 0.11, respectively), and extrathyroidal extension was at a critical level in terms of an association with *TERT* promoter mutations (*P* = 0.03 and 0.06, respectively) [21, 22]. This finding may be explained by the fact that these studies, including the data on focus numbers of vascular invasion and extrathyroidal extension, were relatively small. Consistently, one finding in the studies by Yin et al. [21] and Liu et al. [22] and our study was that *TERT* promoter mutations were not associated with multifocality. To the best of our knowledge, there were no studies showing that *TERT* promoter mutations were associated with multifocality. The previous meta-analyses studies and our meta-analysis conformably demonstrated that patients with *TERT* promoter mutations in PTC were more likely to experience persistence or recurrence, and *TERT* promoter mutations more likely gave rise to mortality for patients with PTC. Therefore, we concluded that *TERT* promoter mutations are responsible for more aggressive clinicopathological features and may represent a poor prognostic factor in PTC. However, the poor prognosis in patients with PTC may be affected also by treatment factors, such as type of surgery, iodine-131 (I^131^) ablation, and the use of external radiotherapy. Therefore, different approaches may be used for their clinical management. More invasive treatment strategies, such as total thyroidectomy or central lymph node dissection, may be considered in patients with PTC presenting *TERT* promoter mutations to decrease recurrence or mortality.

In FTC, the average frequency of *TERT* promoter mutations was 15.1%, which was higher than that in PTC. The majority of the studies reported *TERT* promoter mutations were not detected in FTA [16, 67, 69, 71]. However, a current case report study showed *TERT* promoter (C228T) mutation in a patient with FTA [70]. In addition, another study reported positive *TERT* promoter (C228T) mutations in four (5.3%) of 76 adenomas included in that study, but three of them were atypical follicular thyroid adenomas (AFTA) [26], which are now classified as follicular tumor of uncertain malignant potential by the World Health Organisation (WHO) 2017 guidelines [72]. The single case with *TERT* promoter-mutated FTA later developed scar recurrence and died of FTC [26]. Thus, they concluded that *TERT* promoter mutations may occur as an early genetic event in thyroid follicular tumors that have not developed malignant features on routine histopathological workup. However, this unexpected finding of *TERT* promoter mutations in FTA has rarely been reported, and further studies with larger sample sizes are needed to detect the gene mutation and explain the mechanism. Therefore, this conclusion should be interpreted cautiously.

Furthermore, our study showed that there was no association between *TERT* promoter mutations and most aggressive clinicopathological characteristics in FTC, such as larger tumor size, male gender, vascular invasion, extrathyroidal extension, and LNM, which differed from the results of PTC. This difference might be related to the relatively small sample of FTC. The current study suggested that *TERT* promoter mutations more likely tended to present in older patients with FTC, and were only associated with distant metastasis and advanced TNM stage, but not with gender, tumor size, multifocality, vascular invasion, extrathyroidal extension, and LNM. With the exception of the study by Song et al. [20], which showed that *TERT* promoter mutations were not associated with age, the other results of association between *TERT* promoter mutations and clinicopathological characteristics of FTC were consistent with those of the studies by Wang et al. [26], Muzza et al. [30], and Song et al. [20]. Therefore, there are differences between PTC and FTC in the contributions of *TERT* promoter mutations to clinicopathological features. However, it is coincident that there are strongly association between *TERT* promoter mutations and distant metastasis (OR = 6.15, 6.49, and 24.29, respectively) and advanced TNM stage (OR = 5.68, 4.82, and 5.10, respectively) in DTC, PTC, and FTC. Compared with other clinicopathological features, the *TERT* promoter mutations possibly make more contribution to distant metastasis and advanced TNM stage of DTC, especially to distant metastasis of FTC (OR = 24.29). As described in nearly all the relevant literature on prognosis [16, 20, 26, 67], our meta-analysis showed that *TERT* promoter mutations were strongly associated with persistence/recurrence, and disease-specific mortality, and indicated that patients with *TERT* promoter mutations in FTC also have poor prognosis. Thus, *TERT* promoter mutations may be considered biomarkers for prognosis in FTC. However, more valuable studies on a large cohort of cases are required to evaluate the clinical behavior in patients with FTC.

There were some limitations in this meta-analysis. First, most studies were designed retrospectively, which may cause potential selection bias to better-documented patients and larger tumors, since they were more available for collection and analysis. Second, heterogeneity was present in some analyses probably due to confounding factors, such as patient demographics, ethnicity, sample source, therapeutic approaches, duration of follow-up, and so forth. Furthermore, most of the aggressive variables are interrelated. For example, patients with more advanced disease tend to have LNM and, thus, disease stage may confound the association between *TERT* promoter mutations and LNM. Lastly, the sample sizes of some included articles are relatively small (especially in FTC), and relevant unpublished data could not be obtained for further analysis. Therefore, our conclusions should be interpreted cautiously.

## Conclusion

In conclusion, this meta-analysis demonstrated that *TERT* promoter mutations were likely to present in older patients and were strongly associated with distant metastasis, advanced tumor stage, disease persistence/recurrence, and disease-specific mortality in DTC, and also were associated with male gender, larger tumor size, vascular invasion, extrathyroidal extension, and LNM in PTC, but not in FTC. Therefore, DTC with *TERT* promoter mutations present aggressively clinical behaviors, and *TERT* promoter mutations could be considered as biomarkers assisting in risk stratification, prognostic prediction, and individualizing therapeutic options for DTC (PTC and FTC). However, more and further studies are needed to evaluate the role of *TERT* promoter mutations in FTC.

## Supplementary information

Supplementary Figure S1

Supplementary Figure S2

Supplementary Figure S3

Supplementary Figure S4

Supplementary Figures Legends
