# Supplementary material for: Association between TERT promoter mutations and clinical behaviors in differentiated thyroid carcinoma: a systematic review and meta-analysis
Source: Endocrine. 2019 Oct 26;67(1):44–57. doi: 10.1007/s12020-019-02117-2 (PMC6969012; doi:10.1007/s12020-019-02117-2)
Supplement: Supplementary file 5 — Supplementary Figures Legends [file 12020_2019_2117_MOESM5_ESM.docx]

**Supplemental figure S1** Forest plot showing the association of *TERT* promoter mutations with clinical parameters in PTC

**Supplemental figure S2** Forest plot showing the association of *TERT* promoter mutations with clinical parameters in FTC
